# A phylogenetic approach to study the origin and evolution of the CRINKLY4 family

**DOI:** 10.3389/fpls.2015.00880

**Published:** 2015-10-23

**Authors:** Natalia Nikonorova, Lam D. Vu, Nathan Czyzewicz, Kris Gevaert, Ive De Smet

**Affiliations:** ^1^Department of Plant Systems Biology, Flanders Institute for Biotechnology (VIB)Ghent, Belgium; ^2^Department of Plant Biotechnology and Bioinformatics, Ghent UniversityGhent, Belgium; ^3^Department of Medical Protein Research, Flanders Institute for Biotechnology (VIB), Ghent UniversityGhent, Belgium; ^4^Department of Biochemistry, Ghent UniversityGhent, Belgium; ^5^Division of Plant and Crop Sciences, School of Biosciences, University of NottinghamLoughborough, UK; ^6^Center for Plant Integrative Biology, University of NottinghamLoughborough, UK

**Keywords:** CR4, WOX5, CLV1, CLE40, stem cells, evolution, phylogenetics, crinkly repeat

## Abstract

Cell–cell communication plays a crucial role in plant growth and development and relies to a large extent on peptide ligand–receptor kinase signaling mechanisms. The CRINKLY4 (CR4) family of receptor-like kinases is involved in a wide range of developmental processes in plants, including mediating columella stem cell identity and differentiation in the *Arabidopsis thaliana* root tip. Members of the CR4 family contain a signal peptide, an extracellular part, a single-pass transmembrane helix and an intracellular cytoplasmic protein kinase domain. The main distinguishing features of the family are the presence of seven “crinkly” repeats and a TUMOR NECROSIS FACTOR RECEPTOR (TNFR)-like domain in the extracellular part. Here, we investigated the evolutionary origin of the CR4 family and explored to what extent members of this family are conserved throughout the green lineage. We identified members of the CR4 family in various dicots and monocots, and also in the lycophyte *Selaginella moellendorffii* and the bryophyte *Physcomitrella patens*. In addition, we attempted to gain insight in the evolutionary origin of different CR4-specific domains, and we could detect “crinkly” repeat containing proteins already in single celled algae. Finally, we related the presence of likely functional CR4 orthologs to its best described signaling module comprising CLAVATA3/EMBRYO SURROUNDING REGION-RELATED 40 (CLE40), WUSCHEL RELATED HOMEOBOX 5 (WOX5), CLAVATA 1 (CLV1), and ARABIDOPSIS CR4 (ACR4), and established that this module likely is already present in bryophytes and lycophytes.

## Introduction

Stem cells are fundamental for post-embryonic plant growth, and the stem cell niche is subject to a tight regulation (ten Hove et al., 2015). In plants, stem cells are located in the central zone of the shoot apical meristem and around the quiescent center in the root apical meristem. Initiated during early embryogenesis they constantly produce cells during the entire plant life (Scheres, 2007). Maintaining the balance between cell proliferation, specification, and differentiation requires the coordinated activity of, for example, hormones (Pacifici et al., 2015), peptides (Murphy et al., 2012), (mobile) transcription factors (Han et al., 2014), and receptor kinases (De Smet et al., 2009). Despite the obvious involvement of receptor kinases in animals, our understanding of their role in plant growth and development remains very incomplete.

Over 600 receptor-like kinase genes have been identified in *Arabidopsis thaliana* and similar or even higher numbers were found in other plant species, such as *Physcomitrella patens* (329), soybean (605), tomato (647), rice (977–1132), and poplar (1192) (Shiu and Bleecker, 2001b; Shiu et al., 2004; Lehti-Shiu et al., 2009; Liu et al., 2009). Mutations in receptor kinases often lead to obvious and crucial developmental defects as they are, for example, involved in root and shoot apical meristem maintenance (De Smet et al., 2009; Stahl and Simon, 2012; Wierzba and Tax, 2013).

One of these receptor kinases is ARABIDOPSIS CRINKLY4 (ACR4), which belongs to the CRINKLY4 (CR4) family of receptor-like kinases. CR4 was first identified in maize (*Zea mays*), where the *cr4* mutation affects leaf epidermis differentiation (Becraft et al., 1996). Epidermal cells of maize *cr4* mutants have irregularities in shape, cell wall thickness and structure, cuticle formation, and vesicle trafficking, and often display tumor-like sections with large undifferentiated and disorganized cells (Becraft et al., 1996, 2001; Jin et al., 2000). The *cr4* mutant plants exhibit very short stature and also display defects in floral organs, including the glumes, anthers, and silks, and CR4 functions in aleurone formation in seeds and may be involved in sex-determination (Becraft et al., 1996; Jin et al., 2000; Kang et al., 2002; Tian et al., 2007). Expression studies indicated that the maize *cr4* transcript is present in vegetative and floral organs, but absent in the root (Jin et al., 2000; Becraft et al., 2001; Kang et al., 2002). Following the initial characterization in maize, orthologs of *CR4* were identified and characterized in rice (*Oryza sativa*) and *A. thaliana* (Shiu and Bleecker, 2001b; Cao et al., 2005). Rice *cr4* mutants also show tumor-like cell growth in the outer epidermis, wart-like cell masses in the inner epidermis of palea and lemma, and abnormal cells with discontinuous cuticles and uneven cell walls (Pu et al., 2012). In *A. thaliana, ACR4* is strongly expressed in the protodermal cells of the embryo, in the L1 layer of the shoot apical meristem, in the epidermis of leaf primordia, in the small daughter cells after the first asymmetric pericycle cell division and in the root stem cell niche (Tanaka et al., 2002; Gifford, 2003; Watanabe et al., 2004; De Smet et al., 2008). Subcellular localization of transiently expressed *ACR4* in *Nicotiana benthamiana* leaf epidermal cells shows that ACR4 preferentially associates with plasmodesmata (Stahl et al., 2013). The *ACR4* expression pattern corresponds with *A. thaliana acr4* mutant phenotypes, such as seed development, embryo morphogenesis, various abnormalities related to epidermal differentiation—which include disorganized cell layers in the ovule integument—, loss of coordination of pericycle cell divisions during lateral root initiation, and irregular columella stem cell division and differentiation in the root apex (Gifford, 2003; Watanabe et al., 2004; De Smet et al., 2008).

In the *A. thaliana* genome four CRINKLY4-RELATED (CCR) proteins were found (Cao et al., 2005). Members of the CR4 family contain a signal peptide, an extracellular part, a single-pass transmembrane helix, and a cytoplasmic serine/threonine protein kinase domain (Jin et al., 2000). Kinase assays demonstrated that ACR4 is an active serine/threonine kinase, while CCR1 and CCR2 are nearly inactive in autophosphorylation assays (Gifford, 2003; Cao et al., 2005; Meyer et al., 2011, 2015). The distinguishing feature of the family is the presence of seven “crinkly” repeats in the extracellular part which is required both for signaling and for normal protein internalization (Gifford et al., 2005). A core C(X~10)CWG sequence motif is highly conserved amongst the repeats, and the regularly spaced Cys residues in the extracellular “crinkly” repeat domain are likely to form stabilizing disulfide bridges. Another feature of the CR4 family extracellular part is the presence of a domain with homology to the three Cys-rich repeats of the TUMOR NECROSIS FACTOR RECEPTOR (TNFR) extracellular domain (Becraft et al., 1996; Gifford et al., 2005). Finally, ACR4 and CCRs can interact through the conserved “KD/ESAF” motif in the juxtamembrane domain (Meyer et al., 2013, 2015).

The initial idea that the CR4 ligand may be a peptide (Becraft et al., 1996) was supported in *A. thaliana*, since the *acr4* mutant is largely insensitive to CLAVATA3/EMBRYO SURROUNDING REGION-RELATED 40 (CLE40) peptide application with respect to columella stem cell phenotypes (Stahl et al., 2009). In addition, ACR4 and CLAVATA 1 (CLV1) form homo- and heterodimers that regulate root meristem maintenance in response to the CLE40 signaling peptide (Stahl et al., 2013). Thus, ACR4, CLV1, CLE40 peptide, and the downstream WUSCHEL RELATED HOMEOBOX 5 (WOX5) transcription factor were proposed as a signaling module controlling the stem cell niche in root meristems (Stahl et al., 2009). The involvement of the latter is further supported by the fact that the intracellular domain of ACR4 could interact with and phosphorylate WOX5 (Meyer et al., 2015).

Here, we investigated the phylogenetic relationships between CR4 family members in the green plant lineage and explored when members of this family (or key motifs) appeared. In addition, we searched for putative orthologs of other components of the CLE40-ACR4-CLV1-WOX5 signaling module in the green plant lineage.

## Materials and methods

### Data sources and candidate retrieval

In our analysis we included genomes of 23 species of single-celled (*Ostreococcus lucimarinus, Ostreococcus tauri, Micromonas* sp. RCC299, *Micromonas pusilla* CCMP1545, *Chlorella vulgaris, Chlorella variabilis*, and *Chlamydomonas reinhardtii*), colony-forming (*Volvox carteri*), and multicellular green algae (*Spirogyra pratensis* and *Coleochaete orbicularis*), the bryophyte *Physcomitrella patens*, the lycophyte *Selaginella moellendorffii* and various higher plants, including the monocots *Sorghum bicolor, Zea mays, Hordeum vulgare, Triticum aestivum*, and *Orysa sativa* and the dicots *Populus trichocarpa, Cucumis sativus, Medicago truncatula, Glycine max, Vitis vinifera*, and *Solanum tuberosum*. The *A. thaliana* genome encodes ACR4 and four CRINKLY4-RELATED (CCR) proteins (Cao et al., 2005). We used the respective *A. thaliana* full-length protein sequences as well as their extracellular domain sequences (Supplementary data set 1) for BLASTP analyses on www.uniprot.org, http://blast.st-va.ncbi.nlm.nih.gov/Blast.cgi, and www.phytozome.net with an *E*-value = 1 as filter, and retrieved a large number of putative CR4 family members (data not shown). Subsequently, reciprocal BLASTP analyses (where we BLASTed the highest-scoring candidate protein sequence against an organism database with well-annotated and described proteins) were performed for putative CR4 family members against the *A. thaliana* protein database TAIR10 Proteins (www.arabidopsis.org/Blast/index.jsp). Putative CR4 orthologs were considered when the protein of interest was retrieved in the top five for the reciprocal BLASTP analyses in *A. thaliana*. A similar approach was used for CLE40, WOX5, and CLV1 (Supplementary data set 1). The complete workflow is summarized in Supplementary Figure 1.

### Alignment and phylogenetic analyses

Retrieved sequences were aligned using a progressive alignment algorithm (Feng and Doolittle, 1987) in order to create multiple sequence alignments with CLC DNA Workbench 7 using the following settings: Gap open cost (10), Extension cost (1), End gap cost (as any other), Alignment (very accurate). To visualize conserved motifs and domains we also used WebLogo (weblogo.berkeley.edu; Crooks, 2004). The phylogenetic trees for the *A. thaliana* CR4 family and for Viridiplantae were built using the Neighbor Joining and UPGMA tree construction method, respectively. For protein distance measurement a Jukes–Cantor method was used and a bootstrap analysis with 1000 replicates within the CLC DNA Workbench 6 (CLC Bio-Qiagen, Aarhus, Denmark) was performed. Putative CR4 orthologs were considered further when they grouped in the clade of the protein of interest and matched to the results obtained after the reciprocal BLASTP analysis. The taxonomy tree was generated with the phyloT online tool (phylot.biobyte.de).

### Domain prediction

For the prediction of signal peptides, the PSORT prediction tool for plants (http://psort.hgc.jp/form.html) together with the SignalP 4.1 server (www.cbs.dtu.dk/services/SignalP) were used with the standard settings for PSORT and the following parameters for SignalP: organism group (Eukaryotes), D-cutoff values (Default), method (input sequences may include TM regions). For the transmembrane and kinase domain prediction, TMHMM 2.0 (www.cbs.dtu.dk/services/TMHMM) and the Pfam database (pfam.xfam.org/) were used, respectively.

### 3D structure modeling

Structure prediction for the putative extracellular domain of ACR4 from *A. thaliana* (NP_191501.1), *Zea mays* (NP_001105395.1), *Selaginella moellendorffii* (XP_002960988.1), and *Physcomitrella patens* (XP_001754560.1) was carried out in the SWISS-MODEL workspace (http://swissmodel.expasy.org/workspace/; Arnold et al., 2006; Guex et al., 2009; Kiefer et al., 2009). For the same purpose, full length sequences of ACR4 homologs from *Micromonas* sp. RCC299 (XP_002508396.1) and *Chlorella variabilis* (XP_005845729.1) were used. Templates for the modeling studies were identified in automated mode against the SWISS-MODEL template library (Supplementary Table 1). Structure representations were generated using the PyMOL Molecular Graphics System, Version 1.7.4, Schrödinger, LLC (www.pymol.org).

### Data mining

Analyses of available transcriptome data was done through the Genevestigator platform (genevestigator.com/gv/index.jsp; Zimmermann et al., 2004, 2005; Hruz et al., 2008) on 27/07/2015.

## Results and discussion

### Features of the CR4 family in *A. thaliana*

**Figure 1:**
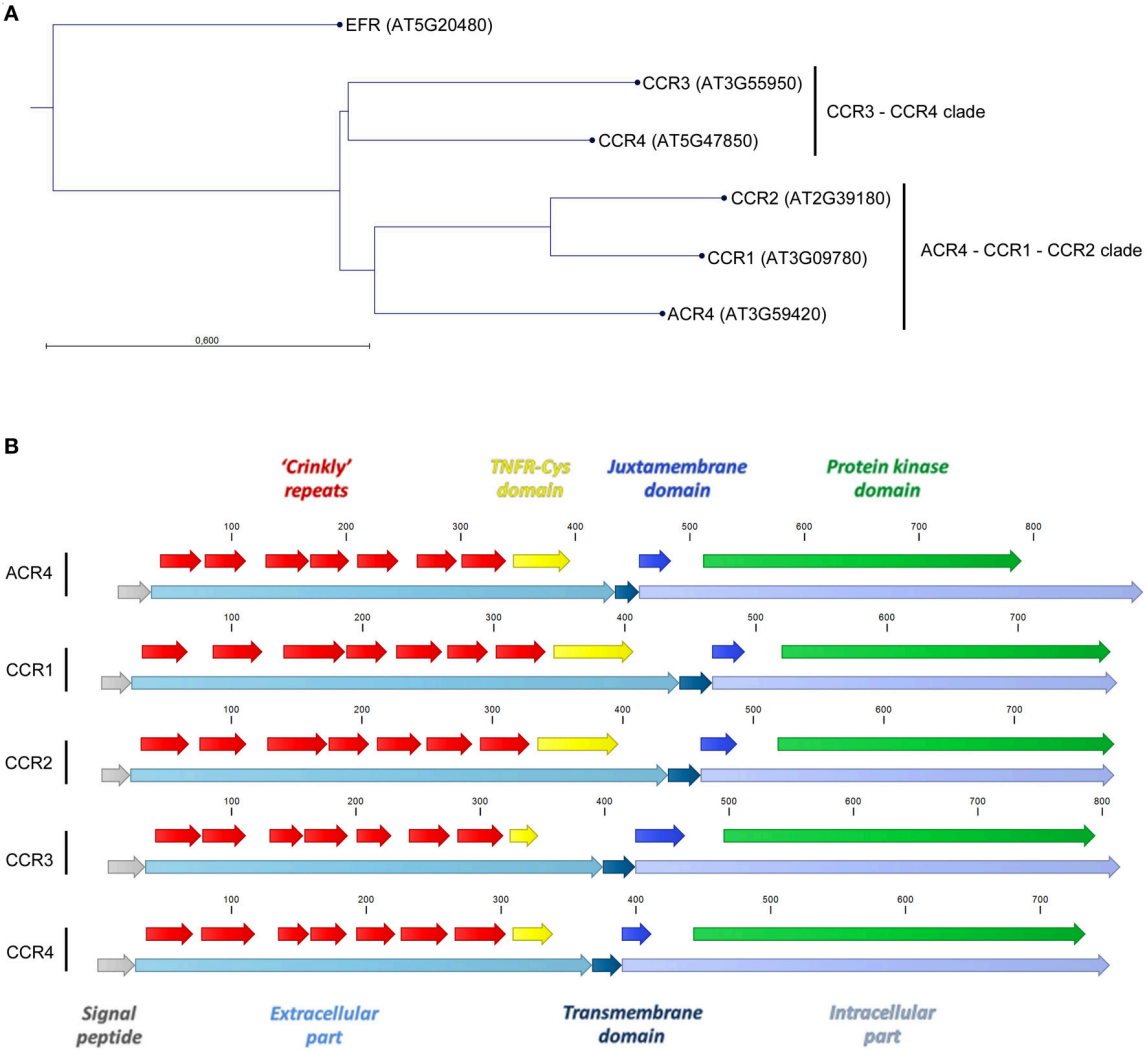
***A. thaliana* CR4 family members. (A)** Neighbor joining tree construction method with Jukes–Cantor protein distance measure and bootstrap analysis with 1000 replicates. **(B)** Common features for *A. thaliana* CR4 family members.

**Figure 2:**
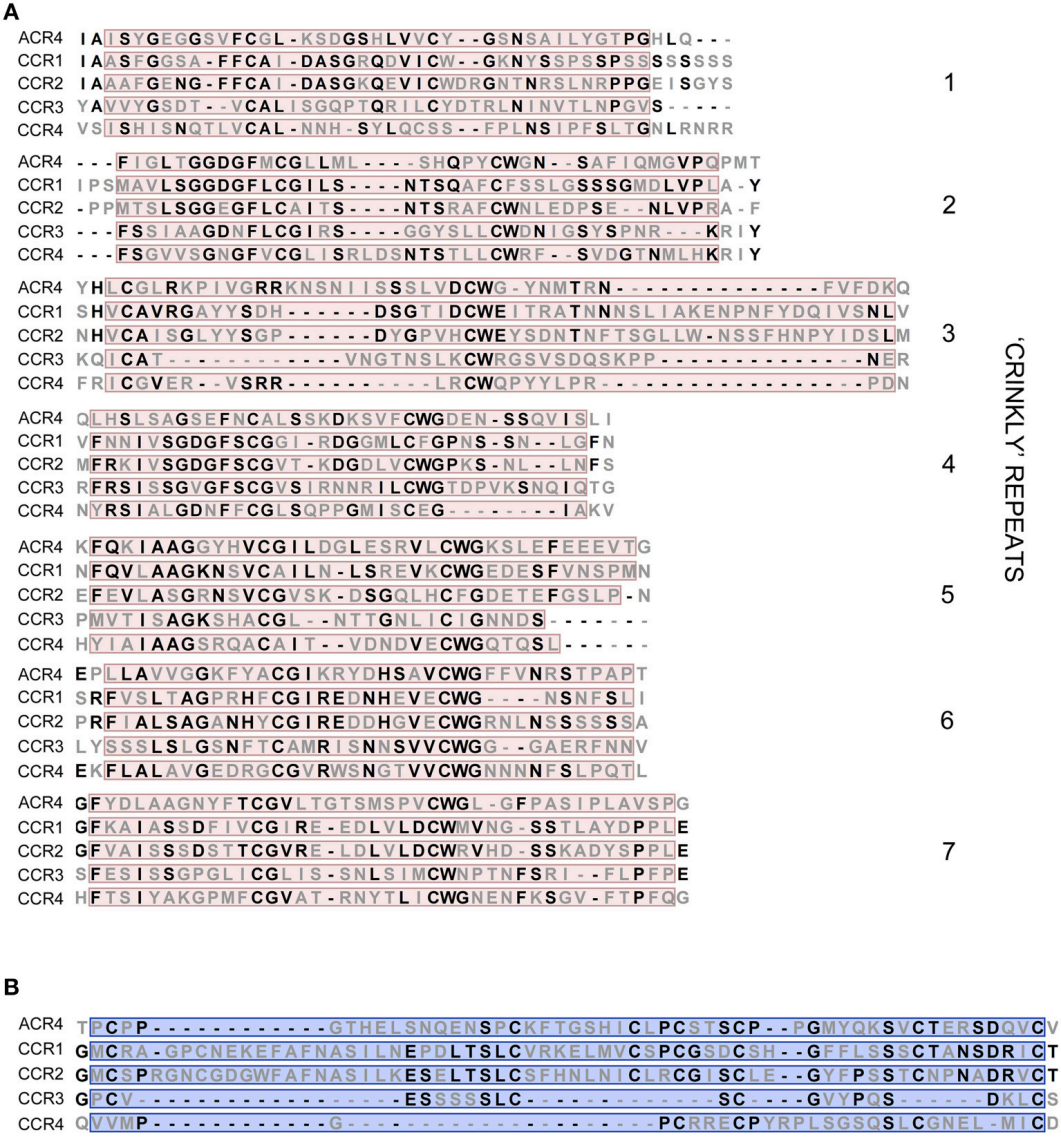
**Key domains in extracellular part of *A. thaliana* CR4 family members. (A,B)** Alignment of amino acid sequences of seven “crinkly” repeats **(A)** and TNFR-Cys domain **(B)** of *A. thaliana* CR4 family members. Conserved residues are in bold.

In *A. thaliana*, the CR4 family consists of five members distributed over two clades; one that includes ARABIDOPSIS CRINKLY 4 (ACR4)/CRINKLY 4 (CR4) (AT3G59420–Q9LX29), CRINKLY 4 RELATED 1 (CCR1 or CRR1) (AT3G09780–Q9S7D9), and CRINKLY 4 RELATED 2 (CCR2 or CRR2) (AT2G39180–O80963) and one with CRINKLY 4 RELATED 3 (CCR3 or CRR3) (AT3G55950–Q9LY50) and CRINKLY 4 RELATED 4 (CCR4)/CRINKLY 4-RELATED KINASE 1 or CYTOKININ-REGULATED KINASE 1 (CRK1) (AT5G47850–Q9FIJ6) (Figure 1A). All the family members share common features, such as a signal peptide, an extracellular part (with characteristic features such as seven “crinkly” repeats and a TNFR-Cys domain), a transmembrane domain and an intracellular part (including a juxtamembrane and protein kinase domain; Figure 1B). The ACR4 extracellular part is required for protein function, and the “crinkly” repeats include several cysteine residues in C(X~10)CWG motifs that are likely important for stabilizing disulfide bridges and generating the β-propeller structure of the extracellular part (Gifford et al., 2005) (Figure 2A). The ACR4 extracellular part also contains a domain with homology to the three Cys-rich repeats of the TNFR extracellular domain (Becraft et al., 1996; Gifford et al., 2005) (Figure 2B). With respect to the kinase domain, it appears that this is not essential for ACR4 (Gifford et al., 2005) and is likely inactive for CCR1 and CCR2 (Gifford, 2003; Cao et al., 2005). Finally, ACR4 and CCRs can interact through the conserved “KD/ESAF” motif in the juxtamembrane domain (Meyer et al., 2013, 2015).

### Identification of putative CR4 family members in the green lineage

While there is some information on CR4 family members in a few plant species, such as *A. thaliana*, maize, and rice (Becraft et al., 1996; Gifford, 2003; Watanabe et al., 2004; Cao et al., 2005; Pu et al., 2012), a comprehensive overview is lacking. In addition, available web-resources such as Plaza (Proost et al., 2009), Phytozome (Goodstein et al., 2012), and Ensembl Plants (Kersey et al., 2014) appeared to be incomplete and do not always apply sufficient criteria to identify true orhologs. To gain insight into the size and evolutionary origin of the CR4 family, we analyzed available genomes from the green plant lineage. In our analysis we included genomes of single-celled, colony-forming and multicellular green algae, the bryophyte *Physcomitrella patens*, the lycophyte *Selaginella moellendorffii*, and higher plants, totaling 23 species. Through extensive BLASTP and reciprocal BLASTP analyses, we generated a set of possible CR4 family members (Supplementary Table 2).

Our analyses showed that putative members of the CR4 family are present in algae, bryophytes, lycophytes, and in all higher plant species selected for our analysis (Supplementary Table 2). Among the 10 algae species included in our search, BLASTP analyses revealed only three putative orthologs of CR4 family proteins encoded in the genomes of *Chlorella variabilis, Micromonas pusilla*, and *Micromonas* sp. RCC299. However, due to the lack of complete genome information for the more multicellular algae, such as *S. pratensis* and *C. orbicularis*, we cannot rule out that CR4 family members are present in those species. In addition, there appeared to be no putative orthologs for CCR1 and CCR2 in monocots, which is consistent with a previous study (Cao et al., 2005). Furthermore, we could not identify putative CCR1 and CCR2 orthologs in *Medicago truncatula*.

As proteins are generally composed of one or more functional regions or domains that can provide insight into their function and evolutionary relationship (Lee et al., 2007), we explored to what extent the key domains and motifs are conserved in the putative CR4 family members (see below). Taking these data into account will allow us to generate a conclusive list of likely functional CR4 family members.

### “Crinkly” repeats and TNFR domain in putative CR4 family members

**Figure 3:**
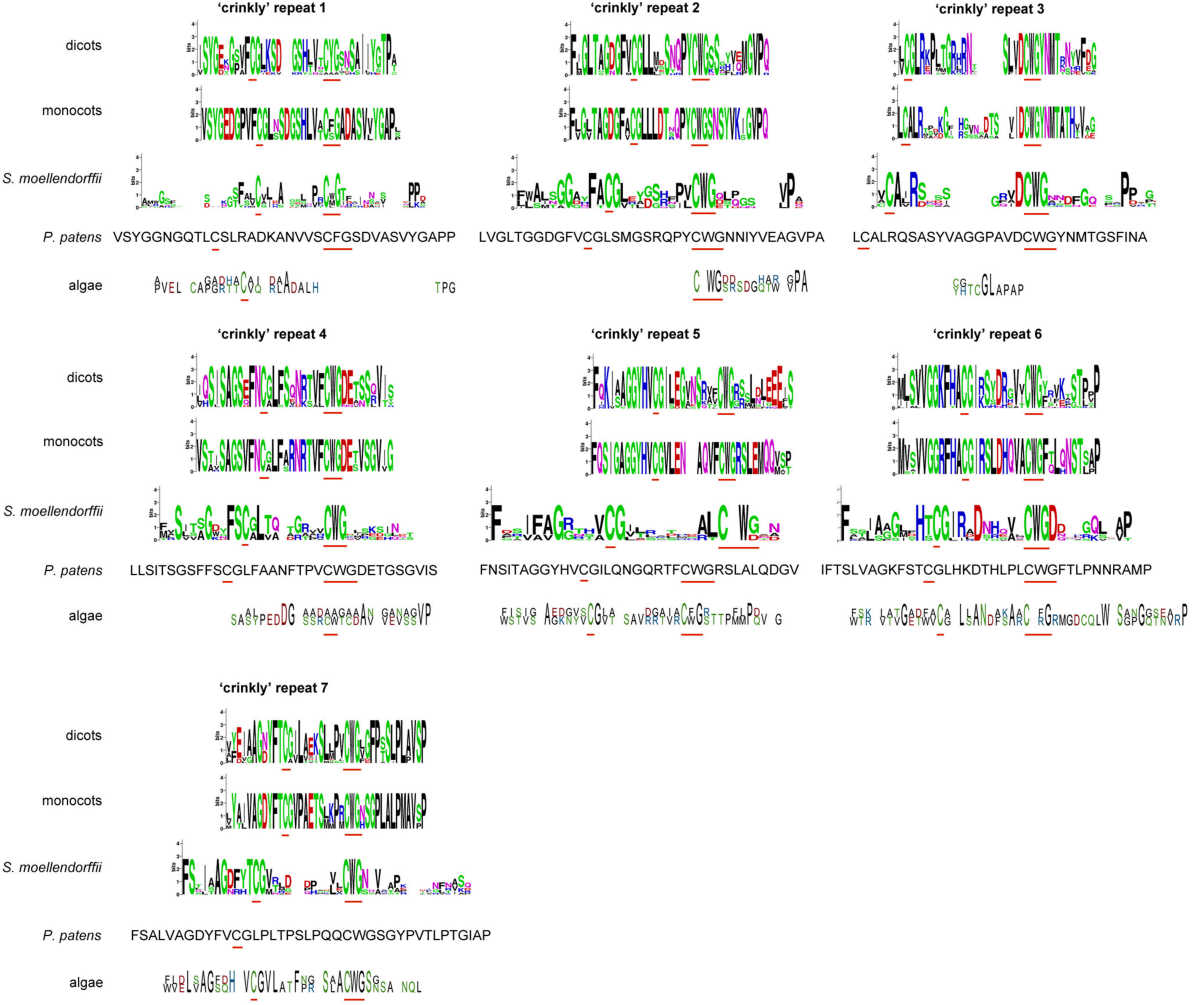
**“Crinkly” repeats sequences of (putative) CR4 family orthologs**. WebLogo or sequence of “crinkly” repeats in dicots *(A. thaliana, Populus trichocarpa, Cucumis sativus, Medicago truncatula, Glycine max, Vitis vinifera, Solanum tuberosum*), monocots (*Sorghum bicolor, Zea mays, Hordeum vulgare, Triticum aestivum, Orysa sativa*), the bryophyte *Physcomitrella patens*, the lycophyte *Selaginella moellendorffii*, and algae (*Chlorella variabilis* and *Micromonas* sp. RCC299). Red lines indicate conserved C(X~10)CWG motif.

**Figure 4:**
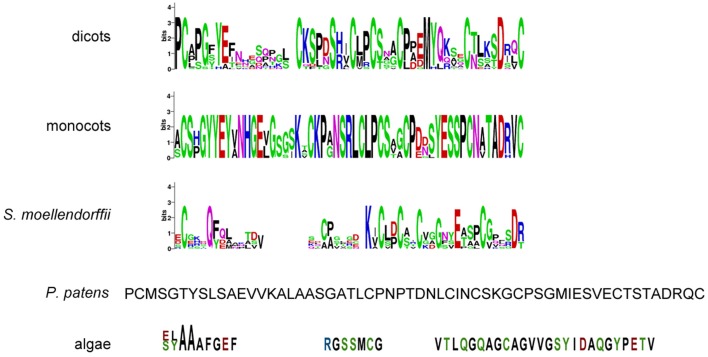
**TNFR domain sequences of (putative) CR4 family orthologs**. WebLogo or sequence of TNFR domain in dicots *(A. thaliana, Populus trichocarpa, Cucumis sativus, Medicago truncatula, Glycine max, Vitis vinifera, Solanum tuberosum*), monocots (*Sorghum bicolor, Zea mays, Hordeum vulgare, Triticum aestivum, Orysa sativa*), the bryophyte *Physcomitrella patens*, the lycophyte *Selaginella moellendorffii*, and algae (*Chlorella variabilis* and *Micromonas* sp. RCC299).

Since the extracellular part is required for protein function and normal protein internalization (Gifford et al., 2005), the set of putative CR4 family members was first investigated in terms of presence and conservation level of key extracellular domains, including “crinkly” repeats and the TNFR domain. Sequence alignment indicated that the extracellular part is highly conserved within monocots and dicots (Figure 3 and Supplementary Table 3). Although putative ACR4 orthologs in algae, *P. patens*, and *S. moellendorffii* have a much lower conservation level, the key residues remained highly conserved within the “crinkly” repeats and TNFR domain. With respect to putative ACR4 orthologs, the first “crinkly” repeat is the most variable, since only two Cys, two Gly, and one Pro residue were conserved and for algae sequences only Pro matched to *A. thaliana* sequence. The other six “crinkly” repeats were relatively more conserved and almost in all algae, *P. patens*, and *S. moellendorffii* sequences the C(X~10)CWG motif was detected in each repeat (Figure 3 and Supplementary Table 3). However, only Cys, Gly, and Trp residues of this motif were found in all of repeats. In ACR4 and its putative orthologs in *P. patens* and *S. moellendorffii*, the TNFR domain included seven Cys residues; however, algae species contained none (*Micromonas* sp. RCC299) or two Cys residues (*Chlorella variabilis*; Figure 4 and Supplementary Table 3). Although the TNFR domain of dicots and monocots is conserved, it has some characteristics that distinguish the taxonomic groups. For example, for putative ACR4 orthologs, the Pro at position 346 in ACR4 is highly conserved in all dicot species, but is replaced by Ala or Ser in monocots. Similarly, the Ser at position 360 in ACR4 is highly variable in dicots and is replaced by Lys in monocots. Interestingly, the Gln at position 383 in ACR4, which is conserved in dicots, is substituted for Glu in monocots and also in *P. patens* and *S. moellendorffii* sequences. At the moment, it is not clear if this has structural implications and would affect functionality.

### 3D structure modeling of selected putative CR4 family members

For ACR4, the extracellular part is predicted to fold into a β-propeller type structure (McCarty and Chory, 2000; Cao et al., 2005; Gifford et al., 2005). In the case of ACR4—given the protein–protein interactions formed by similarly structured proteins—one would expect that the β-propeller formed by the extracellular part would facilitate binding of a ligand, which is presumably of peptide/protein origin (McCarty and Chory, 2000; Gifford et al., 2005) and which would result in the activation of the intracellular kinase domain.

Beta-propellers are tertiary structures formed from a number of tandem repeat domains, forming a tilted disc-like structure similar to that of propeller blades (Chaudhuri et al., 2008). These blade domains are mostly thought to originate from single-bladed ancestor proteins in a number of both pro- and eukaryotic organisms (Kopec and Lupas, 2013). These ancestor proteins were subsequently duplicated multiple times, and further tandem repeats of the blade domain were incorporated, resulting in a functionally diverse range of proteins containing β-propeller domains, with varying numbers of blades (Fülöp and Jones, 1999). Usually, each blade consists of four β-sheets which are arranged antiparallel to each other in a “β-meander,” forming a triangular structure (Baker et al., 1997; Chaudhuri et al., 2008). The blades are themselves arranged in a circular structure, and are slightly tilted, forming propeller-like structures containing between 4 and 10 blades (Chen et al., 2011). In order to form the final propeller structure, β-sheets at N- and C-termini interact with each other, to form a closed circle (Baker et al., 1997; Chaudhuri et al., 2008). A common configuration of closing is the 3+1 structure where one β-sheet is present at the C-terminus, and forms the innermost part of the final blade, and three sheets are contributed by the N-terminus, forming a “velcro closing” mechanism by the formation of hydrogen and/or disulfide bonds along the length of two β-sheets (Neer and Smith, 1996; Chaudhuri et al., 2008). There are variations on this theme amongst proteins containing β-propeller domains, namely velcro closing domains can also be formed from 1+3 and 2+2 β-sheets from N- and C-termini, respectively, and in the case of four-bladed β-propellers, disulfide bridges commonly form between the first and last blade domains, replacing the velcro closing mechanism altogether (Fülöp and Jones, 1999). The resulting propeller structure serves to stabilize the protein, and an array of hydrogen donors and acceptors arranged on the edges of the β-strands simultaneously allows for a diverse range of functions, from protein-protein interactions to enzymatic activity (Neer and Smith, 1996; Baker et al., 1997; Fülöp and Jones, 1999). The active site for the imparted function of β-propeller forming proteins is commonly found in the central “tunnel” region, and substrates are determined by size exclusion and interactions with exposed amino acid side-chains (Neer and Smith, 1996; Baker et al., 1997; Fülöp and Jones, 1999).

CR4-like receptors contain seven copies of a ~39 amino acid repeat that is distantly related to REGULATOR OF CHROMOSOME CONDENSATION 1 (RCC1; Renault et al., 1998). Modeling of the ACR4 extracellular “crinkly” repeats was previously undertaken based on the x-ray structures of two seven-bladed β-propeller folds of the β-LACTAMASE INHIBITORY PROTEIN II) (BLIP-II) from *Streptomyces exfoliates* (Lim et al., 2001) and RCC1 from human (Renault et al., 1998). Molecular modeling studies support the structural homology of the CRINKLY4 domain to the RCC1 family of seven-bladed propeller proteins from three, rather than four, antiparallel β-sheets per blade (McCarty and Chory, 2000; Cao et al., 2005; Gifford et al., 2005). According to the earlier analyses, the regularly spaced Cys residues in the extracellular “crinkly” repeat domain, which are absent in template structures, are held close in space on neighboring antiparallel β-strands within each repeat, and thus, are likely to form stabilizing disulfide bridges which act to stabilize the β-propeller structure in the oxidizing extracellular environment (Gifford et al., 2005).

To gain insight in the 3D structure of the extracellular part of potential CR4 family members, we modeled this part from ACR4 from selected species, such as *A. thaliana, Zea mays, Selaginella moellendorffii, Physcomitrella patens, Chlorella variabilis*, and *Micromonas* sp. RCC299 (Supplementary data set 2). While various methods to predict protein structure exist, including the *ab initio* approach (which predicts the protein structure from the physicochemical parameters based on the protein sequence), or the protein threading approach (which uses a specific energy function to determine and evaluate the fitting between the target sequence and the protein folds in the library; Kelley, 2009; Lee et al., 2009), we employed the structure modeling webserver SWISS-MODEL which uses a comparative homology-based modeling algorithm (Arnold et al., 2006) and does not require a large computational resource and is less time-consuming. Despite the low sequence identity and similarity (Supplementary Table 1), largely due to the unspecific sequence of the loops between the repeats, this method sufficiently provided a first insight in the overall structure of the crinkly repeat domain of CR4 family proteins.

**Figure 5:**
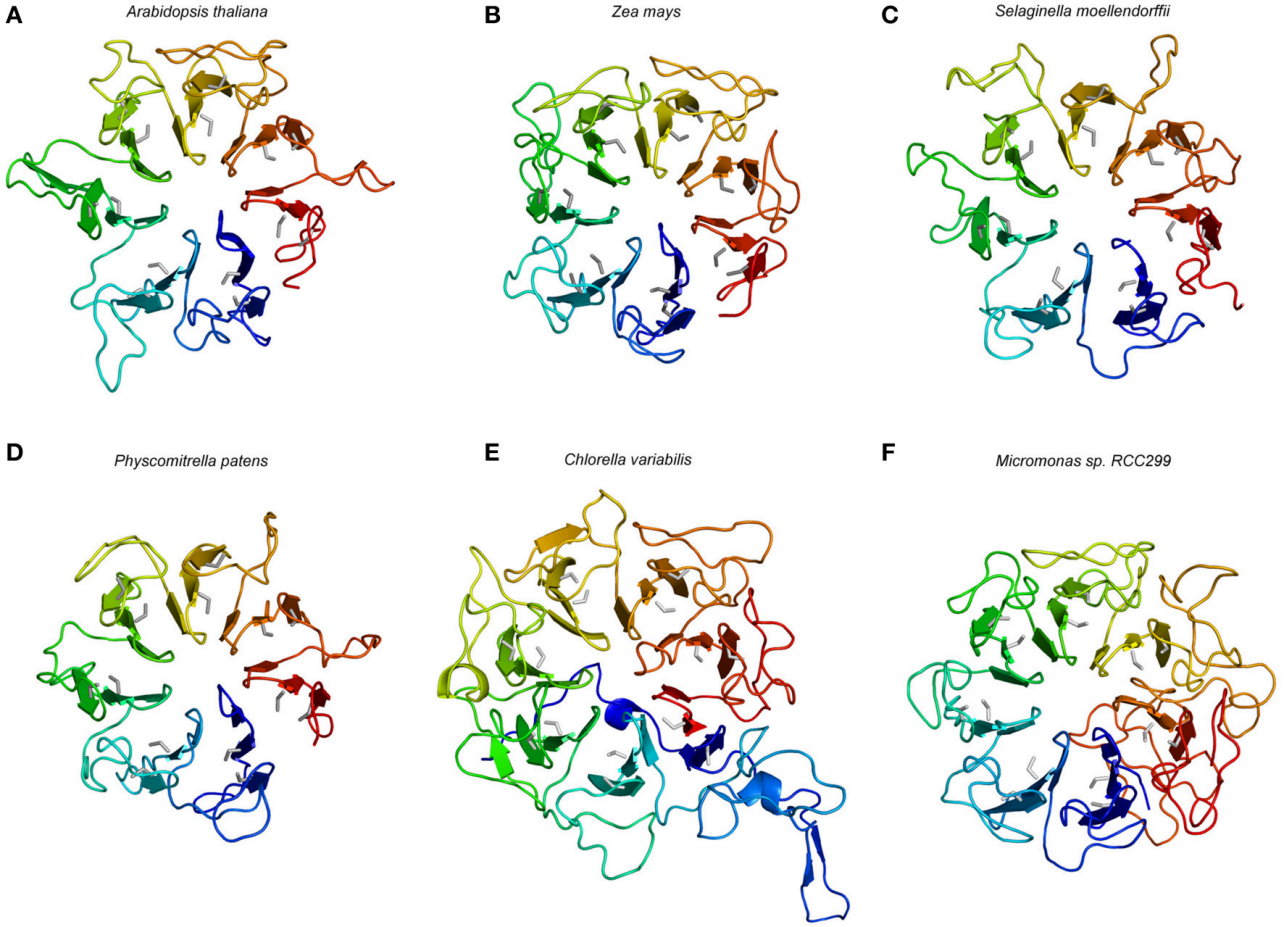
**Structural model of the extracellular part of selected putative CR4 family orthologs**. Models are shown for the dicot *A. thaliana* **(A)**, the monocot *Zea mays* **(B)**, the lycophyte *Selaginella moellendorffii* **(C)**, the bryophyte *Physcomitrella patens* **(D)**, the single-celled algae *Chlorella variabilis* **(E)**, and *Micromonas* sp. RCC299 **(F)**. The color gradient from blue to red represents the (selected) sequence from N-terminus to C-terminus. The conserved cysteine is colored in gray and the seven repeats of blade-shaped β-sheets are visible.

The model structure of the ACR4 extracellular domain was based on the newly available 1.7 Å crystal structure of the plant photoreceptor UV RESISTANCE LOCUS 8 (UVR8) (Christie et al., 2012; also see Materials and Methods). Consistent with the sequence of the protein, the generated ACR4 structure model exhibits an “open” β-propeller fold, similarly to UVR8, with seven structural repeats, each composed of a blade-shaped β-sheet contiguous in sequence (Figure 5). The motifs CGX (X = a hydrophobic amino acid, X = L in the first four repeats, X = I for the next two repeats, and V in the last one, the glycine is exchanged to an alanine the fourth repeat) and CWG (with exception in the first repeat where W is exchange by Y, an amino acid with similar properties) are present in each repeat (Figure 3). These motifs are also found in all the β-bladed repeats of all putative CR4 family orthologs, while not being consistently found in protein structure templates used for the modeling (Figure 3). The predicted disulfide bridges are restrained in the modeled structure. However, the approximate location of the conserved cysteine residues indicates the formation of a disulfide bond, which can be crucial for the folding of the protein and implies the existence of these “crinkly” repeats in putative CR4 family orthologs. An extended sequence in the third repeat of ACR4 is not covered by any similarities with the sequence template used for the modeling and may form an additional loop. Considering their exposed position on the protein surface, the variation in length and composition of these loops across the species may reflect the diversity of the input signaling molecules and may have an impact on the function of the protein. Overall, the structural core elements of *A. thaliana, Z. mays, S. moellendorffii*, and *P. patens* were very similar and suggested a functional extracellular domain (Figures 5A–D).

In the potential CR4 family ortholog from *Chlorella variabilis*, six crinkly repeats with conserved Cys residues are found in the structural model of the retrieved full length protein. Interestingly, a seventh motif with CGV and CWG sub-motifs is found at both ends of the protein sequence, at the N- and C-terminus, respectively. Similar to its template, in this case the human RCC1, the structural model of *C. variabilis* CR4 displays a distinct “velcro closing,” in contrast to other potential CR4 family orthologs that are lacking this feature and represent “open” propeller structures. The structure model also displays longer linking loops between repeats with the greatest length of >30 amino acids (in four loops), compared to 28 (in one loop) in the ACR4 model (Figure 5E). This may indicate distinct structural dynamics and functions of this protein, and since this protein lacks a kinase domain it was not retained as a member of the CR4 family. The potential CR4 family ortholog from *Micromonas* sp. RCC299, of which the model is based on the W285A variant of UVR8, displayed the consistent cysteine-rich motifs and can be considered as the intermediate case between higher plant CR4 and *S. moellendorffii* CR4 (Figure 5F). While representing the seven “crinkly” repeats element and the open propeller structure similarly to the orthologs in higher plant species, the protein is predicted to lack a transmembrane and a kinase domain, and was not retained as a member of the CR4 family.

### Signal peptide, and transmembrane, juxtamembrane, and kinase domains in putative CR4 family members

In addition to the extracellular domain, CR4 family members hold other features that can be used to identify them. We used SignalP 4.1 (Petersen et al., 2011), PSORT (Nakai and Horton, 1999), TMHMM 2.0 (Sonnhammer et al., 1998; Krogh et al., 2001), and the Pfam database (Finn et al., 2014), in addition to sequence alignment, to determine the presence of a signal peptide and transmembrane, juxtamembrane, and kinase domains in putative CR4 family members (Supplementary Table 3).

First, all *A. thaliana* CR4 family members have an N-terminal signal peptide. Commonly signal peptides are 15–30 amino acids long and cleaved off during translocation of a protein across the membrane. The presence of a signal peptide might thus suggest that a protein is targeted to the endoplasmic reticulum and eventually destined to be either secreted, retained in the lumen of the endoplasmic reticulum, lysosome, or any other organelle along the secretory pathway, or to be a single-pass membrane protein (Izard and Kendall, 1994; Mackenzie, 2005). The SignalP 4.1 analysis gave ambiguous results, as for many (potential) CR4 family protein sequences, including *A. thaliana* CCR4, the signal peptide was not found (Supplementary Table 3). Therefore, we also analyzed sequences based on the alignment with annotated *A. thaliana* sequences. This approach allowed us to identify a signal peptide for almost all putative CR4 family members (Supplementary Table 3).

**Figure 6:**
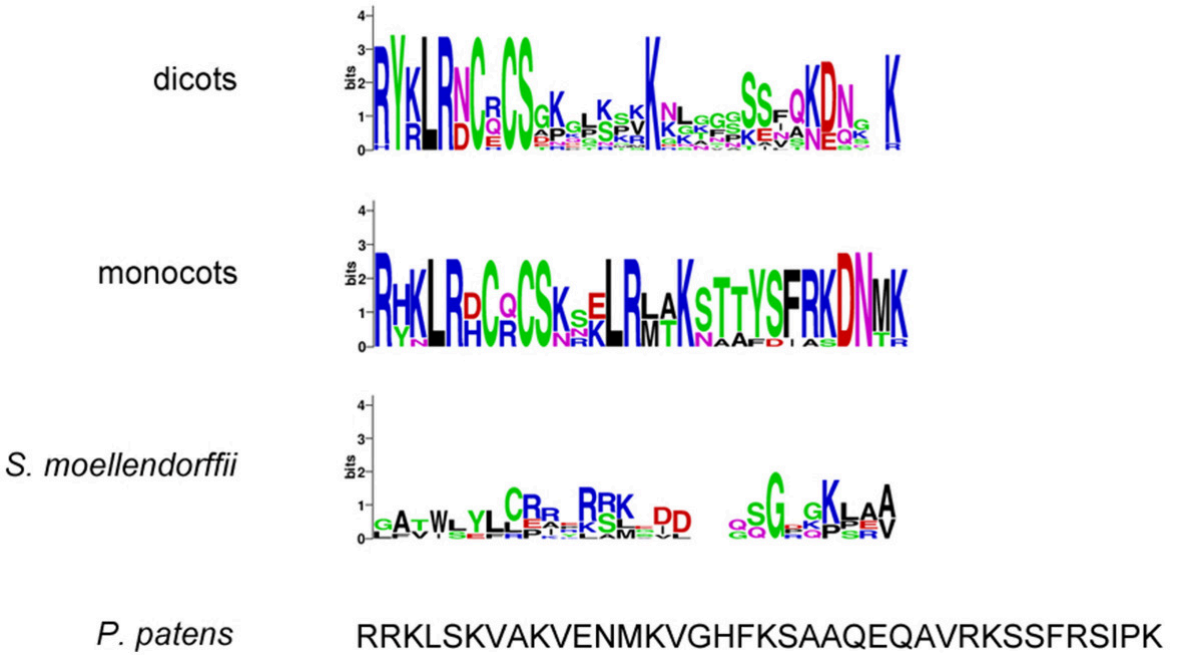
**Juxtamembrane domain sequences of (putative) CR4 family orthologs**. WebLogo or sequence of TNFR domain in dicots *(A. thaliana, Populus trichocarpa, Cucumis sativus, Medicago truncatula, Glycine max, Vitis vinifera, Solanum tuberosum*), monocots (*Sorghum bicolor, Zea mays, Hordeum vulgare, Triticum aestivum, Orysa sativa*), the bryophyte *Physcomitrella patens*, the lycophyte *Selaginella moellendorffii*, and algae (*Chlorella variabilis* and *Micromonas* sp. RCC299).

Second, the highly conserved “KD/ESAF” motif from the juxtamembrane domain within the CR4 family intracellular part is important, for example for protein–protein interaction between family members (Meyer et al., 2015). Our data showed that this motif was not very conserved within species (Figure 6). The total length of this motif in *A. thaliana* is five amino acids, in other dicots nine amino acids and in all monocots seven amino acids. Specifically, only the Lys is highly conserved among both dicots and monocots, the Ala in the ACR4 motif is substituted by Ser in other species, and the Phe is highly conserved only in monocots. In algae, *P. patens* and *S. moellendorffii* a “KD/ESAF” motif could not be detected. The juxtamembrane domain itself is highly conserved in monocots (30 aa), has some variation in dicots (28–32 aa), and is not very conserved in *P. patens* and *S. moellendorffii* (Figure 6 and Supplementary Table 3).

Third, another important feature of CR4 family members is the presence of a 21–26 amino acids long (in *A. thaliana*) transmembrane domain. Our theoretical data based on the TMHMM Server v. 2.0 prediction tool corresponded to the experimental data retrieved from sequence alignments in terms of positioning and length of the domain. In general, transmembrane domains of (potential) CR4 family members consist of 18–30 amino acids. The amino acid composition of transmembrane domains within the CR4 family is different. For example, the transmembrane domain of CCR1, CCR2, and CCR3 subgroups contained 1–2 Cys residue, while no Cys residues were found in CR4 and CCR4. Transmembrane domains are relatively conserved in monocots, dicots, *P. patens* and *S. moellendorffii* but absent in protein sequences of all algae species (*Micromonas sp*., *Micromonas pusilla*, and *Chlorella variabilis*; Supplementary Table 3).

Finally, kinase activity of some members of the CR4 family was demonstrated *in vitro*, and while one report showed that the kinase domain is not essential for ACR4 function, there are other reports suggesting otherwise (Gifford, 2003; Watanabe et al., 2004; Cao et al., 2005; Gifford et al., 2005; Meyer et al., 2011, 2015; Pu and Sun, 2012; Pu et al., 2012). Based on prediction tools and alignment analyses, it could be shown that in most cases a kinase domain was present in the putative CR4 family members. However, almost all candidates from *S. moellendorffii* had no predicted kinase domain except for two protein sequences belonging to the CCR1 clade. Strikingly, a kinase domain was also absent in protein sequences of algae species (*Micromonas sp*. RCC299, *Micromonas pusilla*, and *Chlorella variabilis)*.

### CR4 family members

**Figure 7:**
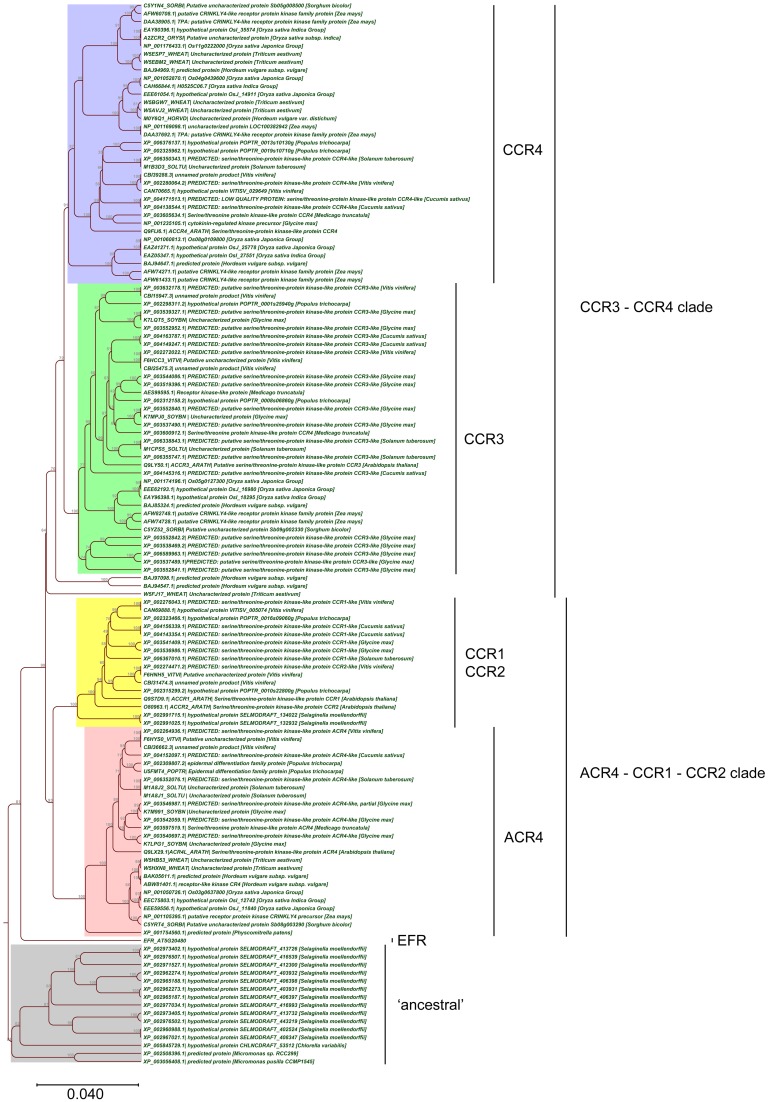
**Phylogenetic tree of CR4 family**. The CR4 family clades are indicated, in addition to the EFR outgroup and possibly proteins “ancestral” to the CR4 family.

**Figure 8:**
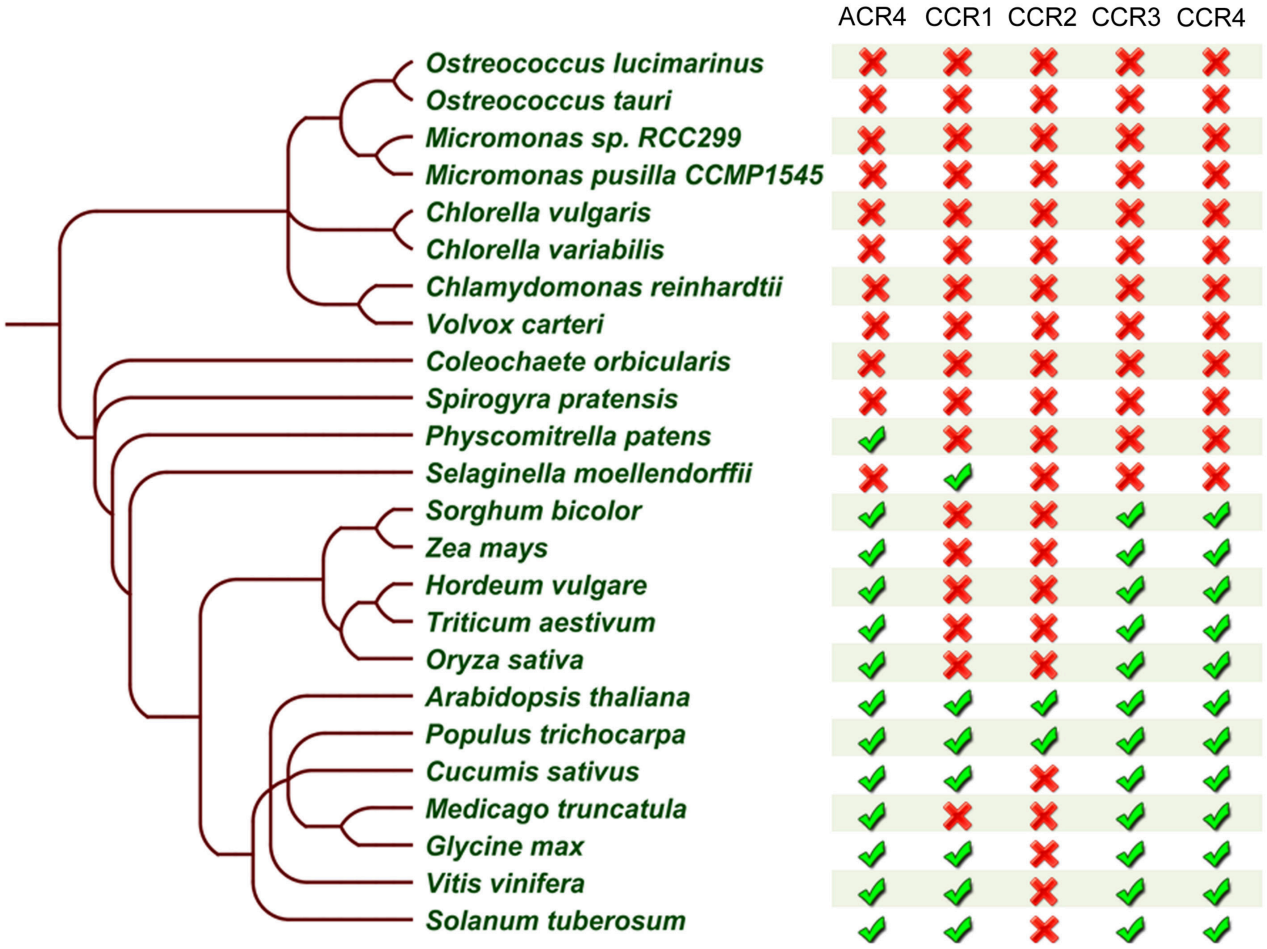
**Summary of CR4 family occurrence in the green lineage**. Table built based on the results after reciprocal BLASTP searches and domain analysis of CR4 family members within selected species. The presence (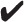) or absence (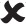) of a likely functional ortholog is indicated.

Taken together, the above-described analyses allowed us to further refine the CR4 family with likely functional orthologs. Overall, land plants contain at least one member of this family (Figures 7, 8), supporting their important role in plant growth and development. Combining the data resulted in a CR4 family tree that consists of two main clades similar to the *A. thaliana* tree. The first clade includes CR4, CCR1, and CCR2 orthologs and the second one includes CCR3 and CCR4 family members. A third, likely evolutionary more ancient one, contains algae and *S. moellendorffii* sequences, mainly potential orthologs of ACR4 and ACCR1 that, however, lack transmembrane and/or kinase domains (Figure 7). For the moss *P. patens* only one protein was found as a true CR4 family member containing all of characteristic domains. At the same time the lycophyte *S. moellendorffii* is represented by two candidates in the ACR4-CCR1-CCR2 clade. Finally, no sequences from algal species were retained based on the necessary presence of all CR4 family characteristics.

Based on the obtained data we concluded that although domains resembling parts of CR4 family members, such as the extracellular part, are present in the investigated non-land plants, these proteins did not appear to act as a receptor kinase on their own. However, due to the lack of complete genome information for multicellular algae, such as *S. pratensis* and *C. orbicularis*, we cannot rule out that CR4 family members are present in those species.

### Evolutionary insight into the CLE40-ACR4-CLV1-WOX5 module

**Figure 9:**
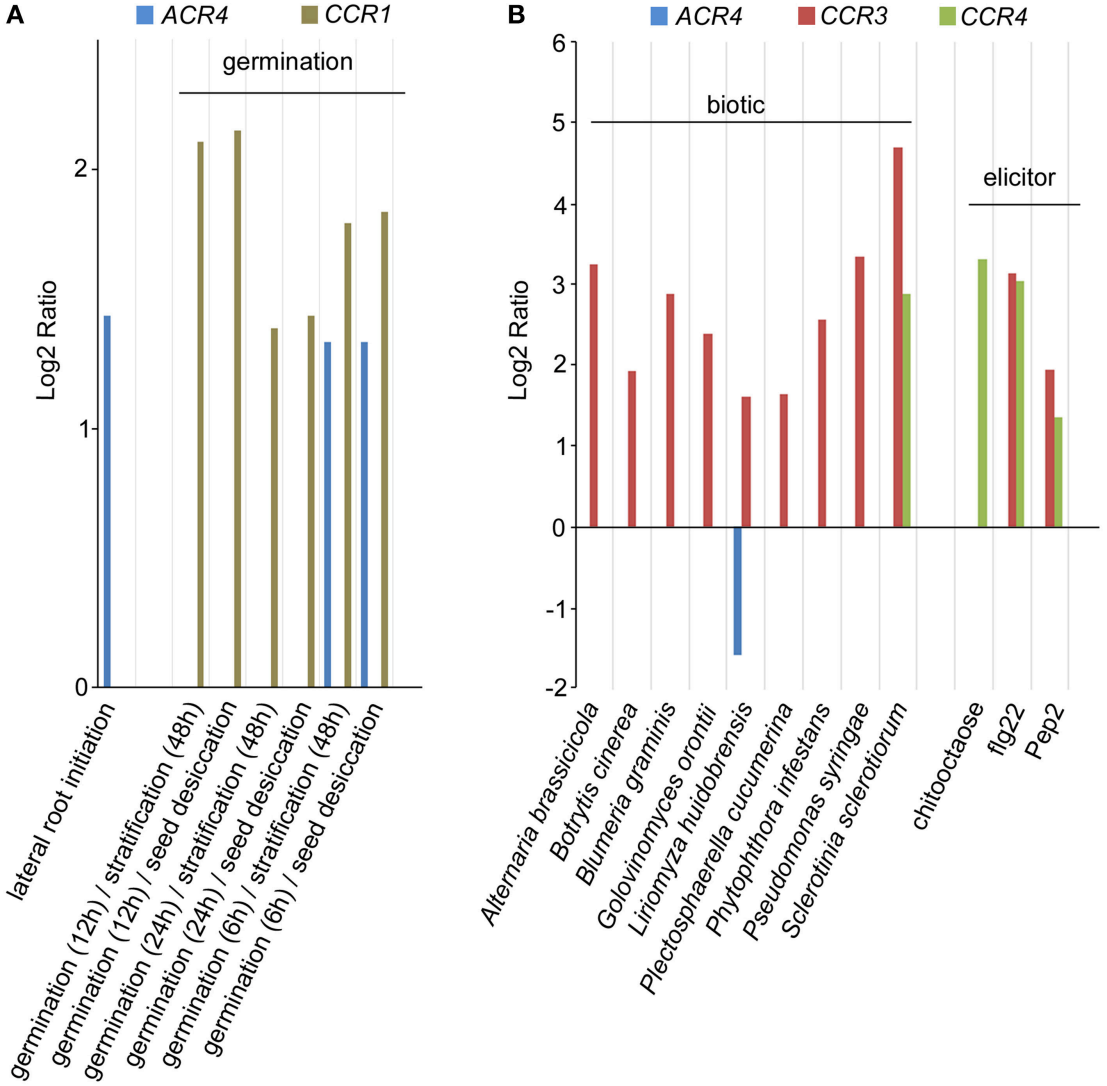
**Expression analysis of *A. thaliana CR4* family. (A,B)** Summary of selected Genevestigator data (Log2 ratio) with respect to lateral root initiation and germination **(A)** and biotic stress and elicitor treatment **(B)** of *CR4* family members in *A. thaliana*. *P* < 0.01. Details on experiments in Supplementary Table 4. Pep2, Elicitor peptide 2; flg22, 22-amino acid sequence of the conserved N-terminal part of flagellin.

While ACR4 has been shown to play important roles in several processes (Becraft et al., 1996; Tanaka et al., 2002; Gifford, 2003; Watanabe et al., 2004; De Smet et al., 2008), the functional characterization of other members of the CR4 family in *A. thaliana* has been hardly addressed, and their characterization is largely limited to a redundant role in lateral root development (De Smet et al., 2008). Given that several receptor kinases—including ACR4—are involved in a multitude of physiological and developmental processes (Shiu and Bleecker, 2001a; De Smet et al., 2009; Stahl and Simon, 2012), it is likely that CR4 family members also play a broader role. To pinpoint new roles for *Arabidopsis* CR4 family members, we probed available micro-array data using Genevestigator (Zimmermann et al., 2004). Based on these results, only *ACR4* expression was found as differentially regulated during the first stages of lateral root initiation (Figure 9A; De Smet et al., 2008). This analysis further revealed that *ACR4* (AT3G59420) and *CCR1* (AT3G09780) are significantly up-regulated during the early stages of seed germination (Figure 9A). In addition, this meta-analysis also revealed a strong regulation of *CR4* family members, specifically *CCR3* and *CCR4*, upon biotic stress and elicitor treatment (Figure 9B). A connection for CR4 family members with biotic stress is further supported by an analysis of the response of *acr4* to *Botrytis cinerea*, which showed that *acr4* has reduced susceptibility to this necrotrophic pathogen (E-Zereen and Ingram, 2012). Furthermore, other environmental and endogenous triggers appeared to regulate expression levels of *CR4* family members in *A. thaliana* (Supplementary Table 4). This information provides interesting leads for future detailed functional characterization.

**Figure 10:**
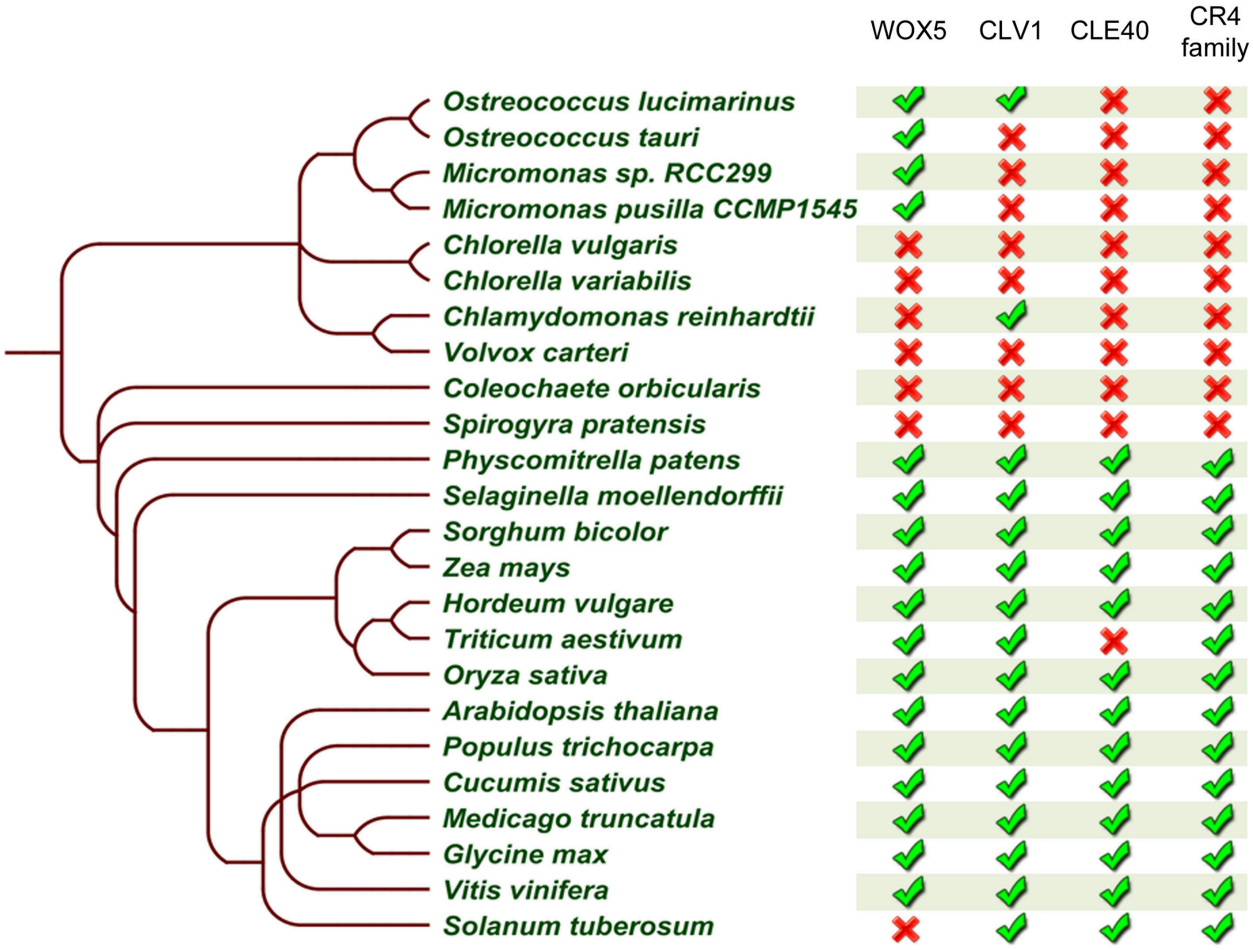
**Summary of CLE40-ACR4-CLV1-WOX5 module occurrence in the green lineage**. Table built based on the results after reciprocal BLASTP searches for WOX5, CLE40, and CLV1 (no further analyses to identify true orthologs), and on a collapse of the CR4 family members. The presence (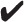) or absence (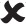) of a likely functional ortholog is indicated. Details can be found in Supplementary Tables 2, 5.

In addition to these observations, our mechanistic understanding of ACR4 action is mainly increasing through the analysis of the CLE40-ACR4-CLV1-WOX5 module in the *A. thaliana* root tip (Stahl et al., 2009, 2013; Meyer et al., 2015; Richards et al., 2015). Therefore, we probed if the other components of this module are also conserved. We performed extensive BLASTP searches and reciprocal BLASTP analyses on the same 23 species included in the CR4 family analysis using *A. thaliana* WOX5, CLV1, and CLE40 sequences as input sequences. This revealed that all components of this signaling module (or closely related proteins) could be identified in *P. patens, S. moellendorffii* and almost all higher plant species except for *Triticum aestivum* and *Solanum tuberosum*. With respect to the latter two, this might be due to incomplete genome sequence information and/or the function of missing components might be replaced by another protein or peptide (Figure 10 and Supplementary Table 5). It is noteworthy that some of the components of this module are present in algae species, but no CLE40 orthologs were identified in these algae species. Taken together, a complete CLE40-ACR4-CLV1-WOX5 module could already be present in *P. patens*, and this can be explored in future.

## Conclusion

Recent studies showed that in monocot and dicot plants, such as *Arabidopsis*, rice, and maize, CR4 receptor kinases play important roles (Becraft et al., 1996; Jin et al., 2000; Tanaka et al., 2002; Gifford, 2003; Watanabe et al., 2004; De Smet et al., 2008; Pu et al., 2012). Given how important CR4 receptor kinases are for root growth, floral organ formation, and seed development, our detailed analysis of this family in a wider range of (crop) plants, could impact on crop improvement. In addition, our results suggest that the origin of the CR4 family coincides with the colonization of terrestrial habitats, as the first CR4 family members with all key domains appear in *P. patens* and *S. moellendorffii*. In future, the function in these species will have to be investigated in detail to reveal if CR4 family members already play a key role in regulating e.g., formative cell divisions. On the other hand, in algae we found ancestral forms of CR4 family members, namely without kinase domain, which could indicate a different role of these proteins.

It is important to note that incomplete genome annotation in some species might explain the absence of some key players. We expect that continuous improvement in genome sequencing and gene/protein annotation will, in future, further increase our insight in the CR4 family and the associated signaling module. In this context, as this is a bio-informatics study, it remains essential to perform functional analyses of the orthologs proposed in this study in order to correctly annotate them and to avoid mis-annotations or over-confident annotations.

### Conflict of interest statement

The authors declare that the research was conducted in the absence of any commercial or financial relationships that could be construed as a potential conflict of interest.
