# Supplementary material for: A phylogenetic approach to study the origin and evolution of the CRINKLY4 family
Source: Front Plant Sci. 2015 Oct 23;6:880. doi: 10.3389/fpls.2015.00880 (PMC4617170; doi:10.3389/fpls.2015.00880)
Supplement: Supplementary file 1 [file DataSheet1.PDF]

Supplementary Material

A phylogenetic approach to study the origin and evolution of the CRINKLY4 family

Natalia Nikonorova[1,2], Lam Dai Vu[1,2,5,6], Nathan Czyzewicz[3], Kris Gevaert[5,6], Ive De Smet[1,2,3,4,*]

[1]Department of Plant Systems Biology, VIB, B–9052 Ghent, Belgium.

[2]Department of Plant Biotechnology and Bioinformatics, Ghent University, B–9052 Ghent, Belgium.

[3]Division of Plant and Crop Sciences, School of Biosciences, University of Nottingham, Loughborough LE12 5RD, United Kingdom

[4]Centre for Plant Integrative Biology, University of Nottingham, Loughborough LE12 5RD, UK

[5]Department of Medical Protein Research, VIB, 9000 Ghent, Belgium

[6]Department of Biochemistry, Ghent University, 9000 Ghent, Belgium

1. Supplementary Data

Supplementary data set 1. *Arabidopsis thaliana* sequences used for the BLAST analyses.

Full-length sequences of Arabidopsis CR4 family members

>sp|Q9LX29|ACR4L_ARATH Serine/threonine-protein kinase-like protein ACR4 OS=Arabidopsis thaliana GN=ACR4 PE=1 SV=1

```
MRMFETRAREWILLVKLVLFTSIWQLASALGSMSSIAISYGEGGSVFCGLKSDGSHLVVCYGSNSAIL
YGTPGHLQFIGLTGGDGFMCGLLMLSHQPYCWGNSAFIQMGVPQPMTKGAEYLEVSAGDYHLCGLRKP
IVGRRKNSNIISSSLVDCWGYNMTRNFVFDKQLHSLSAGSEFNCALSSKDKSVFCWGDENSSQVISLI
PKEKKFQKIAAGGYHVCGILDGLESRVLCWGKSLEFEEEVTGTSTEEKILDLPPKEPLLAVVGGKFYA
CGIKRYDHSAVCWGFFVNRSTPAPTGIGFYDLAAGNYFTCGVLTGTSMSPVCWGLGFPASIPLAVSPG
LCIDTPCPPGTHELSNQENSPCKFTGSHICLPCSTSCPPGMYQKSVCTERSDQVCVYNCSSCSSHDCS
SNCSSSATSGGKEKGKFWSLQLPIATAEIGFALFLVAVVSITAALYIRYRLRNCRCSENDTRSSKDSA
FTKDNGKIRPDLDELQKRRRARVFTYEELEKAADGFKEESIVGKGSFSCVYKGVLRDGTTVAVKRAIM
SSDKQKNSNEFRTELDLLSRLNHAHLLSLLGYCEECGERLLVYEFMAHGSLHNHLHGKNKALKEQLDW
VKRVTIAVQAARGIEYLHGYACPPVIHRDIKSSNILIDEEHNARVADFGLSLLGPVDSGSPLAELPAG
TLGYLDPEYYRLHYLTTKSDVYSFGVLLLEILSGRKAIDMHYEEGNIVEWAVPLIKAGDINALLDPVL
KHPSEIEALKRIVSVACKCVRMRGKDRPSMDKVTTALERALAQLMGNPSSEQPILPTEVVLGSSRMHK
KSWRIGSKRSGSENTEFRGGSWITFPSVTSSQRRKSSASEGDVAEEEDEGRKQQEALRSLEEEIGPAS
PGQSLFLHHNF
```

>sp|Q9S7D9|ACCR1_ARATH Serine/threonine-protein kinase-like protein CCR1 OS=Arabidopsis thaliana GN=CCR1 PE=1 SV=1

```
METRCSLLFLSLILLYLPKPGSGFGSSGPIAASFGGSAFFCAIDASGRQDVICWGKNYSSPSSPSSSS
SSSSIASSTSASYNIPSMAVLSGGDGFLCGILSNTSQAFCFSSLGSSSGMDLVPLAYRTTAYSQIAAG
NSHVCAVRGAYYSDHDSGTIDCWEITRATNNNSLIAKENPNFYDQIVSNLVFNNIVSGDGFSCGGIRD
GGMLCFGPNSSNLGFNTTSDNFQVLAAGKNSVCAILNLSREVKCWGEDESFVNSPMNDSRFVSLTAGP
RHFCGIREDNHEVECWGNSNFSLIPKGSGFKAIASSDFIVCGIREEDLVLDCWMVNGSSTLAYDPPLE
LCSPGMCRAGPCNEKEFAFNASILNEPDLTSLCVRKELMVCSPCGSDCSHGFFLSSSCTANSDRICTP
CSLCQNSSCSDICKLHNSNFPDKHWHQLQRLVLIIGSCASALLIIIIGCCVVPRIVTSPNKEDGAANQ
FKSCIGKPDLDTDQPLENVSPAPSVTPFAQVFRLSELKDATNGFKEFNELGRGSYGFVYKAVLADGRQ
VAVKRANAATIIHTNTREFETELEILCNIRHCNIVNLLGYSTEMGERLLVYEYMPHGTLHDHLHSGFS
PLSWSLRIKIAMQTAKGLEYLHNEAEPRIIHGDVKSSNVLLDSEWVARVADFGLVTSSNEKNLDIKRD
VYDFGVVLLEILTGRKRYDRDCDPPEIVEWTVPVIREGKAAAIVDTYIALPRNVEPLLKLADVAELCV
REDPNQQPTMSELANWLEHVARDALIF
```

>sp|O80963|ACCR2_ARATH Serine/threonine-protein kinase-like protein CCR2 OS=Arabidopsis thaliana GN=CCR2 PE=1 SV=1

```
MQPNSHIFVIITISSLIITVSAYGSTGTIAAAFGENGFFCAIDASGKQEVICWDRGNTNRSLNRPPGE
ISGYSPPMTSLSGGEGFLCAITSNTSRAFCWNLEDPSENLVPRAFQYNSYLQIASGNNHVCAISGLYY
SGPDYGPVHCWEYSDNTNFTSGLLWNSSFHNPYIDSLMFRKIVSGDGFSCGVTKDGDLVCWGPKSNLL
NFSNNEEFEVLASGRNSVCGVSKDSGQLHCFGDETEFGSLPNRPRFIALSAGANHYCGIREDDHGVEC
WGRNLNSSSSSSAPNTSGFVAISSSDSTTCGVRELDLVLDCWRVHDSSKADYSPPLELCSPGMCSPRG
NCGDGWFAFNASILKESELTSLCSFHNLNICLRCGISCLEGYFPSSTCNPNADRVCTPCSLCQNSSCY
GICKIRATKSKEHEQKEQREVRRLVIIIGCSVLGFLVMLIGLSFIPKMTKGSKRDDEERSKMTCCFCF
DKNSVEADPDPVPHQSVLLPTAVSLGETKIFRLSELKDATHGFKEFNELGRGSFGFVYKAVLSDGIHV
AVKRANAATIIHSNNRGFESELEILCKIRHNNIVNLLGYCSEMGERLLVYEYMPHGTLHDHLHGDLSQ
LDWSMRLKIMLQAARGLDYLHNEVDPPIIHRDVKTSNILLDGEMCARIADFGLVSSNERDSSNSDREG
DVYDFGIVLLEILSGRKAIDRESDPAGIAEWAVPLIRKGKAAAIIDRNICLPRNVEPLLKLAELAELA
VRENSNERPNIRNILCFLDLIVKSGLTF
```

>sp|Q9LY50|ACCR3_ARATH Putative serine/threonine-protein kinase-like protein CCR3 OS=Arabidopsis thaliana GN=CCR3 PE=2 SV=1

MKRFINSTVTFSVTVTIAVIIFFLLSPVTSLGSGSTYAVVYGSDTVCALISGQPTQRILCYDTRLNIN
VTLNPGVSFSSIAAGDNFLCGIRSGGYSLLCWDNIGSYSPNRKRIYQNDNVLLETLSVGDKQICATVN
GTNSLKCWRGSVSDQSKPPNERFRSISSGVGFSCGVSIRNNRILCWGTDPVKSNQIQTGFGNTPMVTI
SAGKSHACGLNTTGNLICIGNNDSGQLNVIAPDQPNLYSSSLSLGSNFTCAMRISNNSVVCWGGGAER
FNNVTDSISFESISSGPGLICGLISSNLSIMCWNPTNFSRIFLPFPEVLPGPCVESSSSSLCSCGVYP
QSDKLCSGTGSICKSCPIQFPASPPSQFPLPPPPPPPPPSPSTSSPPSKALTRGLLAFAIVGSVGAFA
GICSVVYCLWTGVCLGKKKVHNSVQPTITRGGSNSRSNSSNSRSLSIRRQGSRMLSMRRQRSGTSSMK
HADKAEEFSFSELASATGNFSLENKIGSGSFGVVYRGKLNDGREVAIKRGEVNAKMKKFQEKETAFDS
EIAFLSRLHHKHLVRLVGYCEEREEKLLVYDYMKNGALYDHLHDKNNVEKHSSLINSWKMRIKIALDA
ARGIEYLHNYAVPPIIHRDIKSSNILLDSNWVARVSDFGLSLMGPVLGKDHNPYQRPTKAAGTVGYID
PEYYSLNVLTDKSDVYGLGVVLLELLTGKRAIFRNNGDVEEEEGCVPVHLVDYSVPAITADELSTILD
PRVGSPELGEGDAVELVAYTAMHCVNAEGRNRPTMTDIVGNLERALDLCGDSHGSISSGICSIVSD

>sp|Q9FIJ6|ACCR4_ARATH Serine/threonine-protein kinase-like protein CCR4 OS=Arabidopsis thaliana GN=CCR4 PE=1 SV=1

MALTISISCFSSYFVSLLLLVLSSFSFVCFSLSTVSISHISNQTLVCALNNHSYLQCSSFPLNSIPFS
LTGNLRNRRFSGVVSGNGFVCGLISRLDSNTSTLLCWRFSVDGTNMLHKRIYHGPELEELEAGNFRIC
GVERVSRRLRCWQPYYLPRPDNYRSIALGDNFFCGLSQPPGMISCEGIAKVPSGDHYIAIAAGSRQAC
AITVDNDVECWGQTQSLPREKFLALAVGEDRGCGVRWSNGTVVCWGNNNNFSLPQTLKDIHFTSIYAK
GPMFCGVATRNYTLICWGNENFKSGVFTPFQGLISQVVMPGPCRRECPYRPLSGSQSLCGNELMICDL
KRNDGEFPDTRAQNSKNKTWSRRNIAFLVVGCVGTFSLLLVISFLIFKSHCRCRVHDSGRLDDTRTID
IPKLEKRLCTLASLGNPGQLMEFSIDELALATDGFSVRFHLGIGSFGSVYQGVLSDGRHVAIKRAELT
NPTLSGTTMRHRRADKDSAFVNELESMSRLNHKNLVRLLGFYEDTEERILVYEYMKNGSLADHLHNPQ
FDPLSWQTRLMIALDAARGIQYLHEFIVPPVIHRDIKSSNILLDATWTAKVSDFGLSQMGPTEEDDVS
HLSLHAAGTLGYIDPEYYKFQQLTTKSDVYSFGVVLLELLSGHKAIHNNEDENPRNLVEYVVPYILLD
EAHRILDQRIPPPTPYEIEAVAHVGYLAAECLMPCSRKRPSMVEVVSKLESALAACLTAPKTETVSRS
NTY

<u>Extracellular domain sequences of Arabidopsis CR4 family members</u>

>sp|Q9LX29|30-434

LGSMSSIAISYGEGGSVFCGLKSDGSHLVVCYGSNSAILYGTPGHLQFIGLTGGDGFMCGLLMLSHQP
YCWGNSAFIQMGVPQPMTKGAEYLEVSAGDYHLCGLRKPIVGRRKNSNIISSSLVDCWGYNMTRNFVF
DKQLHSLSAGSEFNCALSSKDKSVFCWGDENSSQVISLIPKEKKFQKIAAGGYHVCGILDGLESRVLC
WGKSLEFEEEVTGTSTEEKILDLPPKEPLLAVVGGKFYACGIKRYDHSAVCWGFFVNRSTPAPTGIGF
YDLAAGNYFTCGVLTGTSMSPVCWGLGFPASIPLAVSPGLCIDTPCPPGTHELSNQENSPCKFTGSHI
CLPCSTSCPPGMYQKSVCTERSDQVCVYNCSSCSSHDCSSNCSSSATSGGKEKGKFWSLQLPIAT

>sp|Q9S7D9|24-439

FGSSGPIAASFGGSAFFCAIDASGRQDVICWGKNYSSPSSPSSSSSSSSIASSTSASYNIPSMAVLSG
GDGFLCGILSNTSQAFCFSSLGSSSGMDLVPLAYRTTAYSQIAAGNSHVCAVRGAYYSDHDSGTIDCW
EITRATNNNSLIAKENPNFYDQIVSNLVFNNIVSGDGFSCGGIRDGGMLCFGPNSSNLGFNTTSDNFQ
VLAAGKNSVCAILNLSREVKCWGEDESFVNSPMNDSRFVSLTAGPRHFCGIREDNHEVECWGNSNFSL
IPKGSGFKAIASSDFIVCGIREEDLVLDCWMVNGSSTLAYDPPLELCSPGMCRAGPCNEKEFAFNASI
LNEPDLTSLCVRKELMVCSPCGSDCSHGFFLSSSCTANSDRICTPCSLCQNSSCSDICKLHNSNFPDK
HWHQLQRL

>sp|O80963|23-432

YGSTGTIAAAFGENGFFCAIDASGKQEVICWDRGNTNRSLNRPPGEISGYSPPMTSLSGGEGFLCAIT
SNTSRAFCWNLEDPSENLVPRAFQYNSYLQIASGNNHVCAISGLYYSGPDYGPVHCWEYSDNTNFTSG
LLWNSSFHNPYIDSLMFRKIVSGDGFSCGVTKDGDLVCWGPKSNLLNFSNNEEFEVLASGRNSVCGVS
KDSGQLHCFGDETEFGSLPNRPRFIALSAGANHYCGIREDDHGVECWGRNLNSSSSSSAPNTSGFVAI
SSSDSTTCGVRELDLVLDCWRVHDSSKADYSPPLELCSPGMCSPRGNCGDGWFAFNASILKESELTSL
CSFHNLNICLRCGISCLEGYFPSSTCNPNADRVCTPCSLCQNSSCYGICKIRATKSKEHEQKEQREVR
RL

>sp|Q9LY50|31-393

LGSGSTYAVVYGSDTVCALISGQPTQRILCYDTRLNINVTLNPGVSFSSIAAGDNFLCGIRSGGYSLL
CWDNIGSYSPNRKRIYQNDNVLLETLSVGDKQICATVNGTNSLKCWRGSVSDQSKPPNERFRSISSGV
GFSCGVSIRNNRILCWGTDPVKSNQIQTGFGNTPMVTISAGKSHACGLNTTGNLICIGNNDSGQLNVI
APDQPNLYSSSLSLGSNFTCAMRISNNSVVCWGGGAERFNNVTDSISFESISSGPGLICGLISSNLSI
MCWNPTNFSRIFLPFPEVLPGPCVESSSSSLCSCGVYPQSDKLCSGTGSICKSCPIQFPASPPSQFPL
PPPPPPPPPSPSTSSPPSKALTR

>sp|Q9FIJ6|32-366

LSTVSISHISNQTLVCALNNHSYLQCSSFPLNSIPFSLTGNLRNRRFSGVVSGNGFVCGLISRLDSNT
STLLCWRFSVDGTNMLHKRIYHGPELEELEAGNFRICGVERVSRRLRCWQPYYLPRPDNYRSIALGDN
FFCGLSQPPGMISCEGIAKVPSGDHYIAIAAGSRQACAITVDNDVECWGQTQSLPREKFLALAVGEDR
GCGVRWSNGTVVCWGNNNNFSLPQTLKDIHFTSIYAKGPMFCGVATRNYTLICWGNENFKSGVFTPFQ
GLISQVVMPGPCRRECPYRPLSGSQSLCGNELMICDLKRNDGEFPDTRAQNSKNKTWSRRNIA

<u>Full-length sequences of Arabidopsis CLV1, WOX5, CLE40</u>

>sp|Q9SYQ8|CLV1_ARATH Receptor protein kinase CLAVATA1 OS=Arabidopsis thaliana GN=CLV1 PE=1 SV=3

MAMRLLKTHLLFLHLYLFFSPCFAYTDMEVLLNLKSSMIGPKGHGLHDWIHSSSPDAHCSFSGVSCDD
DARVISLNVSFTPLFGTISPEIGMLTHLVNLTLAANNFTGELPLEMKSLTSLKVLNISNNGNLTGTFP
GEILKAMVDLEVLDTYNNNFNGKLPPEMSELKKLKYLSFGGNFFSGEIPESYGDIQSLEYLGLNGAGL
SGKSPAFLSRLKNLREMYIGYYNSYTGGVPPEFGGLTKLEILDMASCTLTGEIPTSLSNLKHLHTLFL
HINNLTGHIPPELSGLVSLKSLDLSINQLTGEIPQSFINLGNITLINLFRNNLYGQIPEAIGELPKLE
VFEVWENNFTLQLPANLGRNGNLIKLDVSDNHLTGLIPKDLCRGEKLEMLILSNNFFFGPIPEELGKC
KSLTKIRIVKNLLNGTVPAGLFNLPLVTIIELTDNFFSGELPVTMSGDVLDQIYLSNNWFSGEIPPAI
GNFPNLQTLFLDRNRFRGNIPREIFELKHLSRINTSANNITGGIPDSISRCSTLISVDLSRNRINGEI
PKGINNVKNLGTLNISGNQLTGSIPTGIGNMTSLTTLDLSFNDLSGRVPLGGQFLVFNETSFAGNTYL
CLPHRVSCPTRPGQTSDHNHTALFSPSRIVITVIAAITGLILISVAIRQMNKKKNQKSLAWKLTAFQK
LDFKSEDVLECLKEENIIGKGGAGIVYRGSMPNNVDVAIKRLVGRGTGRSDHGFTAEIQTLGRIRHRH
IVRLLGYVANKDTNLLLYEYMPNGSLGELLHGSKGGHLQWETRHRVAVEAAKGLCYLHHDCSPLILHR
DVKSNNILLDSDFEAHVADFGLAKFLVDGAASECMSSIAGSYGYIAPEYAYTLKVDEKSDVYSFGVVL
LELIAGKKPVGEFGEGVDIVRWVRNTEEEITQPSDAAIVVAIVDPRLTGYPLTSVIHVFKIAMMCVEE
EAAARPTMREVVHMLTNPPKSVANLIAF

>sp|Q8H1D2|WOX5_ARATH WUSCHEL-related homeobox 5 OS=Arabidopsis thaliana GN=WOX5 PE=2 SV=1

MSFSVKGRSLRGNNNGGTGTKCGRWNPTVEQLKILTDLFRAGLRTPTTDQIQKISTELSFYGKIESKN
VFYWFQNHKARERQKRRKISIDFDHHHHQPSTRDVFEISEEDCQEEEKVIETLQLFPVNSFEDSNSKV
DKMRARGNNQYREYIRETTTTSFSPYSSCGAEMEHPPPLDLRLSFL

>sp|Q9LXU0|CLE40_ARATH CLAVATA3/ESR (CLE)-related protein 40 OS=Arabidopsis thaliana GN=CLE40 PE=2 SV=1

MAAMKYKGSVFIILVILLLSSSLLAHSSSTKSFFWLGETQDTKAMKKEKKIDGGTANEVEERQVPTGS
DPLHHKHIPFTP

Supplementary data set 2. Input sequences for SWISS-MODEL.

Arabidopsis thaliana (putative extracellular part) – ACR4

```
LGSMSSIAISYGEGGSVFCGLKSDGSHLVVCYGSNSAILYGTPGHLQFIGLTGGDGFMCGLLMLSHQP
YCWGNSAFIQMGVPQPMTKGAEYLEVSAGDYHLCGLRKPIVGRRKNSNIISSSLVDCWGYNMTRNFVF
DKQLHSLSAGSEFNCALSSKDKSVFCWGDENSSQVISLIPKEKKFQKIAAGGYHVCGILDGLESRVLC
WGKSLEFEEEVTGTSTEEKILDLPPKEPLLAVVGGKFYACGIKRYDHSAVCWGFFVNRSTPAPTGIGF
YDLAAGNYFTCGVLTGTSMSPVCWGLGFPASIPLAVSPGLCIDTPCPPGTHELSNQENSPCKFTGSHI
CLPCSTSCPPGMYQKSVCTERSDQVCVYNCSSCSSHDCSSNCSSSATSGGKEKGKFWSLQLPIAT
```

Zea mays (putative extracellular part) – CR4

```
LGSMSSIAVSYGEDGPVFCGLNSDGSHLVACFGADASVLYGAPPNIPFLGLTAGDGFVCGLLLDTRQP
YCWGSNSYVKSGVPQPMVEGARYSELSAGDNHLCALRAAQDGGRGSSAATSLIDCWGYNMTATHAVDE
AVSTVSAGSVFNCGLFARNRTVFCWGDETVSGVVGLAPRDLHFQSIGAGGYHVCGVLENAQVFCWGRS
LEMQQVVPSSAIGDGDVNIVPMDAMSTVVGGRFHACGIRSLDHQVACWGFTLHNSTSPPKGLKMYALV
AGDYFTCGVPAETSLMPRCWGNSGPLALPMAVPPGICVPTACSHGYYEYVNHGEVGSIKVCKPANSRL
CLPCSTGCPEGLYESSPCNATADRVCQFDCLKCVTDECLSFCLSQKRTKSRKLMAFQMRIFV
```

Selaginella moellendorffii (putative extracellular part)

```
FGYMSTMAALWGSGSSPVKSFSVCGIDASSGNPRCWGTFAINNASIPPANISLWSLTGGAYFACGLTL
DGHNPVCWGQTPGSIVPAAFQGVRYSTINCGGWHVCAIRDKSILPGDEGRVDCWGNNDFGQCSPPITI
PMRSITAGDYFSCGLTQYTGRVVCWGQMSGDHNDSAQRNRFNQRSLFDTIAAGRTHVCGILRKDHRAL
CWGANDHGQCSPPEGVAFSAISAGMFHTCGIRLDNHSAQCWGDDSVGQSSAPAGVAFSLITAGDRYTC
GVRLDRDDHGGVECWGNQVAAPPDLGFNASQGVCVESSSCRSTQFELAPLVVVAGDNLTETAATTGCK
ICSPCATCGDGFYEAAACGQSRDTECSSCAA
```

Physcomitrella patens (putative extracellular part)

```
LGSMNSVAVSYGGNGQTLCSLRADKANVVSCFGSDVASVYGAPPRLPLVGLTGGDGFVCGLSMGSRQP
YCWGNNIYVEAGVPAVGDRHYAALSAGDNHLCALRQSASYVAGGPAVDCWGYNMTGSFINAPLLSITS
GSFFSCGLFAANFTPVCWGDETGSGVISTAPKGLEFNSITAGGYHVCGILQNGQRTFCWGRSLALQDG
VPKGAIFTSLVAGKFSTCGLHKDTHLPLCWGFTLPNNRAMPTNVPFSALVAGDYFVCGLPLTPSLPQQ
CWGSGYPVTLPTGIAPGMCSSLPCMSGTYSLSAEVVKALAASGATLCPNPTDNLCINCSKGCPSGMIE
SVECTSTADRQCSYDCSHCNHNATCSAACYNPSKAPRTESIQIPIII
```

Micromonas sp. RCC299 (full length)

```
MTRRAGAVLALALFVVVSPLDRLGVAADSPTLSAGARHTCAIDAADALHCWGDDSDGQTRVPADVAAW
RAVAAAKGGCHTCGLALDGAARCWGCDANGEVSGVPTVPGGNRWISVGAGKGVSCGVAAVDRAVRCWG
RFMPQVGANPWYGPWSRVTVGEDWVCALSANDAKARCRGWSANGQTNVPPSLANAAWLDLSAGFQHVC
GVLATFPRSLACWGSGAAGESTPPTPTEEEMAADPSSSIGSWGAVSAGTHVTCGIVGAQRRLKCWGKA
LDYVPGSEYAAAFGEFGQVAGAADVATWGTIVSSSGGDSSGGACSWDVVATGKHHACAIVAQPSSSSS
GAQYTNDSSNVSSNVDASLRWAAKEPGSTEPGRVVCWGDERGGKTRVPSAGVTLPWRAWPATFDDAVD
VVAPPFGFTAQRTAAVGTCATVSAALGGRSVAGGSGWDVFVSLASATLATAAIALASSR
```

Chlorella variabilis (full length)

MPAPAAVVAAAGASPLVPAPQPAPAEPVELCAPGDTACVQRLATPGAVACWGSRHAWGPAPAEQPAAA
LAASTADDGYGGLAPAPSASYPEDDGSSDAATAAAVVANASVPTPLGSSLVFSTISAEDNYVCGLTSR
GTIACFGSTTPMLPDDPNYLPPTELPFEGPFTKLATGATFACGLLANFSAACFGRMGDCQLWSGPGGS
EARPTPLVVGGGLQFVELVAGSDHVCGVLFNGSAACWGSNSANQLGVDSADVATSLTPIAIADPSLAF
VSLAAADTVSCGTLINASVACWGGYTDASQSELLPPQPRIVGADQIASLAARGSSMCGVTLQGQAGCA
GVVGSYIDAQGYPETVFSEQPTPVPSPASFTTITVGGSGFYFHACGLTLEGAALCWGLNNEGQLGVPP
YEVANSTAAVPVATDIRFSVLSAGGDYTCGVVA

2. Supplementary Figures and Tables

2.1. Supplementary Tables (see separate Excel files)

Supplementary Table 1. Summary of tertiary structure prediction of CR4 family members.

Supplementary Table 2. Set of putative CR4 family members.

Supplementary Table 3. Characterization of key domains in putative CR4 family members.

Supplementary Table 4. Genevestigator output for *Arabidopsis thaliana ACR4*, *CCR1*, *CCR2*, *CCR3* and *CCR4*. Significant Log(2)-ratio values are indicated. P-value < 0.01.

Supplementary Table 5. Set of putative orthologues for components of CLE40−ACR4−CLV1−WOX5 signalling module in *Arabidopsis thaliana*.

2.2. Supplementary Figures

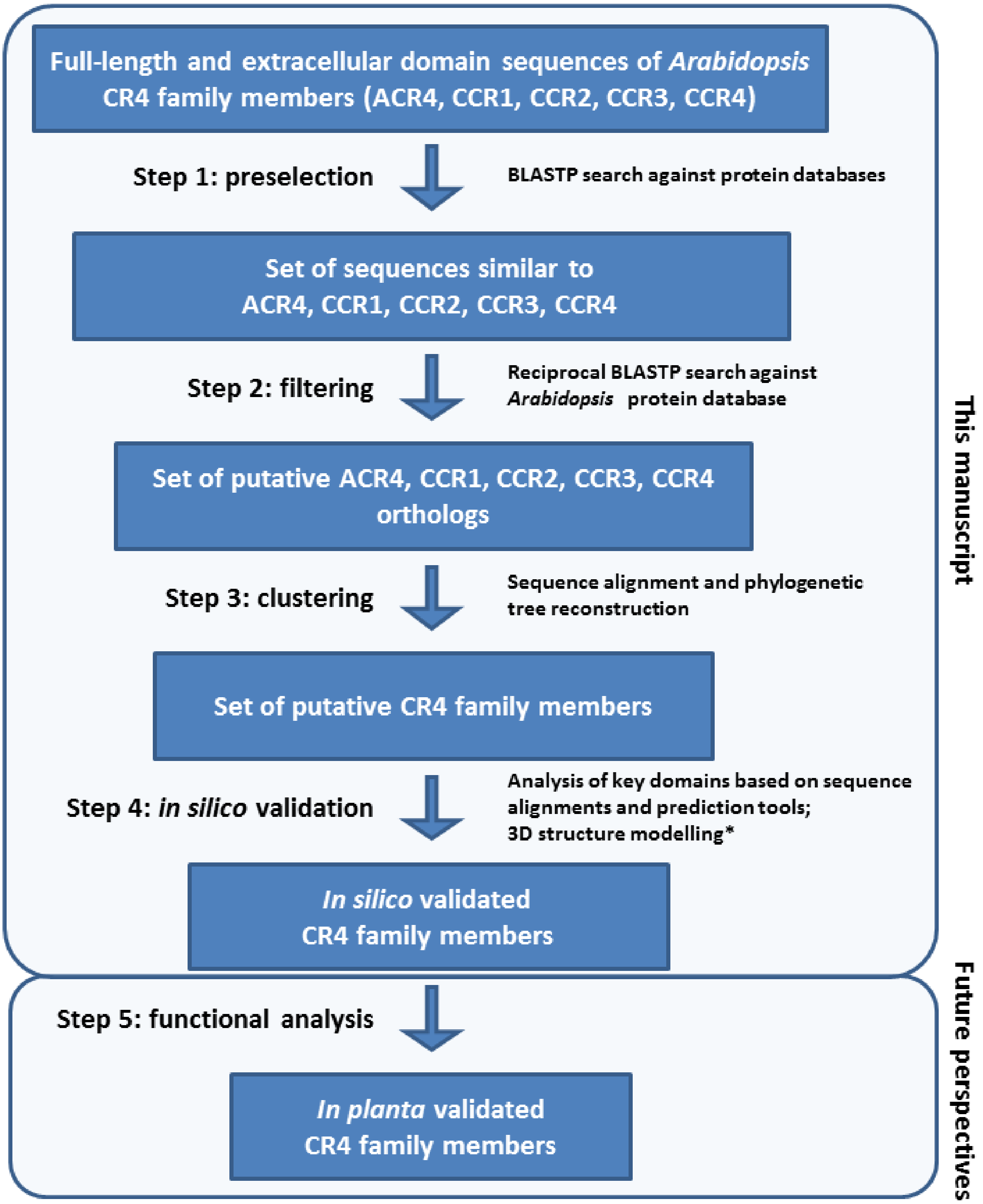

Supplementary Figure 1. General workflow diagram summarizing the bioinformatics approaches used in this study and future perspectives. Asterisk indicates that 3D structure modelling was performed only for selected protein sequences.
